# Supplementary material for: Exploratory Metabolomics and Lipidomics Profiling Contributes to Understanding How Curcumin Improves Quality of Goat Semen Stored at 16 °C in Tropical Areas
Source: Int J Mol Sci. 2024 Sep 23;25(18):10200. doi: 10.3390/ijms251810200 (PMC11432619; doi:10.3390/ijms251810200)
Supplement: Supplementary file 1 [file ijms-25-10200-s001.zip › Supplement Figure.pdf]

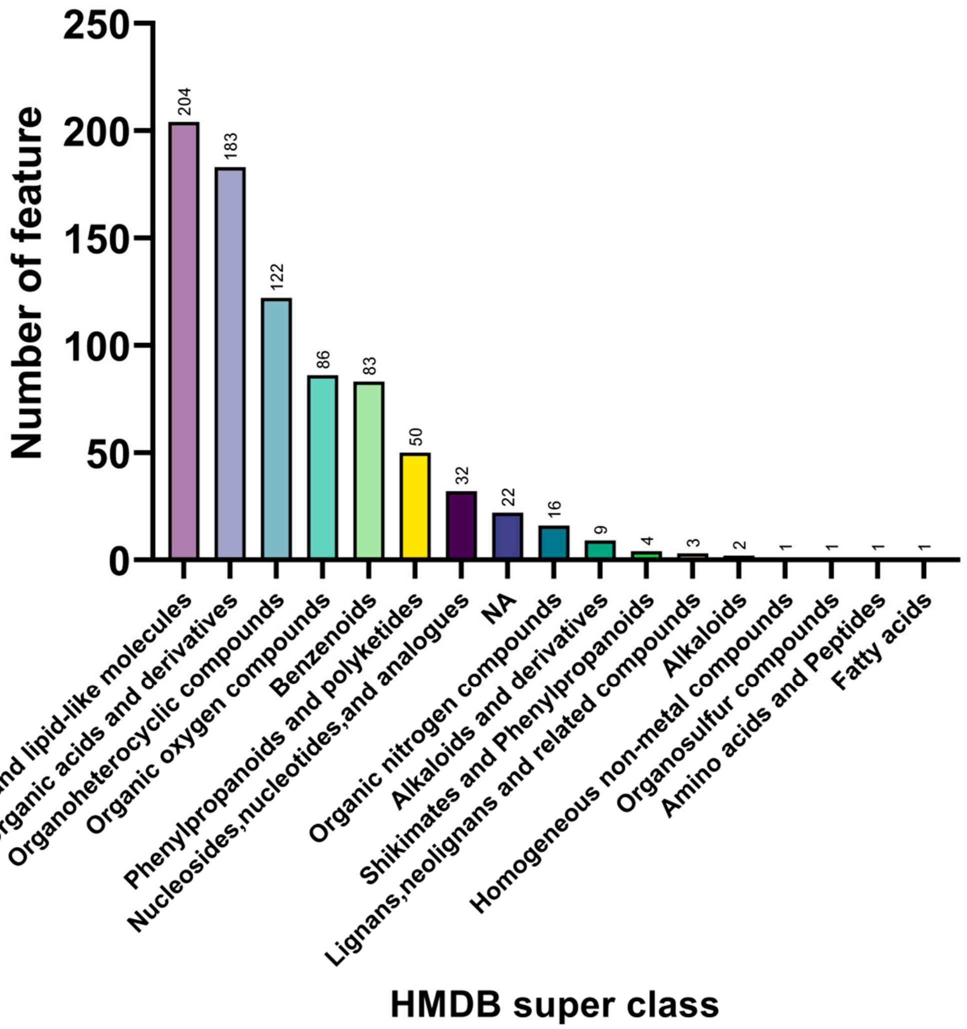

Supplementary Figure 1. Number and classification of the differential metabolites.

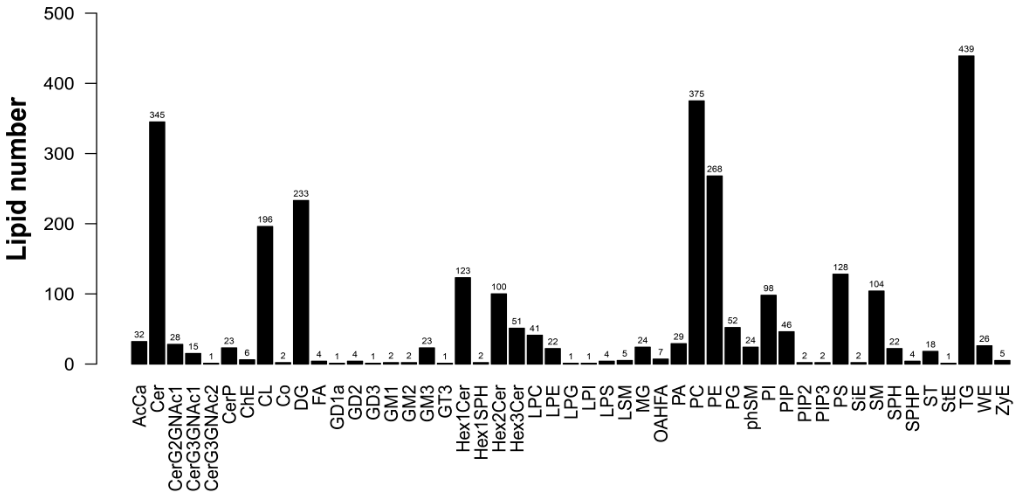

Supplementary Figure 2. Statistics of the lipid subclasses and of the lipid molecules.

Abbreviations: AcCa (acylcarnitine), Cer (ceramide), CerG2GNAc1 (Simple Glc series), CerP (phosphate ceramide), chE (cholesterol lipid), CL (cardiolipin), Co (coenzyme), DG (glycerodiamide), FA (fatty acid), GD (ganglioside), GM3 (ganglioside), Hex1Cer (hexanceramide), SPH (sphingomyelin), LPC (Lysophosphatidyl choline), LPG (dissolved phosphatidylylglycerol), LPI (LP), LPS) (lytic ophosphatidylserine), LSM (LMP), LMG (monoacylglycerol), OAHFA ((O-acyl) -1-hydroxy fatty acid), PA (phosphatidic acid), PE (phosphatidylethanolamine), PC (phosphatidylcholine), PG (phosphatidylglycerol), PhSM (phytosphingosine), PI (phosphatidyllethanol), PIP (phosphatidyllinositol-4-phosphoric acid), PIP2 (phosphatidylinositol-4, 5-diphosphate), PS (phosphatidylserine), SiE (sitotenyl ester), PIP3 (phosphatidylinositol), SM (sphingomyelin phospholipid), ST (sterol lipid), StE (stigmatyl ester), TG (triglycerides), ZyE (yeast sterol), SH (sphingosine), SPHP (sphingosine phosphate).
